# Endoplasmic reticulum stress in human skeletal muscle: any contribution to sarcopenia?

**DOI:** 10.3389/fphys.2013.00236

**Published:** 2013-09-03

**Authors:** Louise Deldicque

**Affiliations:** Exercise Physiology Research Group, Department of Kinesiology, FaBeR, KU LeuvenLeuven, Belgium

**Keywords:** unfolded protein response, anabolic resistance, ageing, exercise, PERK, IRE1α, ATF6

## Abstract

Skeletal muscle is vital to life as it provides the mechanical power for locomotion, posture and breathing. Beyond these vital functions, skeletal muscle also plays an essential role in the regulation of whole body metabolism, e.g., glucose homeostasis. Although progressive loss of muscle mass with age seems unavoidable, it is critical for older people to keep the highest mass as possible. It is clear that the origin of sarcopenia is multifactorial but, in the present review, it was deliberately chosen to evaluate the likely contribution of one specific cellular stress, namely the endoplasmic reticulum (ER) stress. It is proposed that ER stress can: (1) directly impact muscle mass as one fate of prolonged and unresolved ER stress is cell death and; (2) indirectly create a state of anabolic resistance by inhibiting the mammalian target of rapamycin complex 1 (mTORC1) pathway. With age, many of the key components of the unfolded protein response, such as the chaperones and enzymes, display reduced expression and activity resulting in a dysfunctional ER, accelerating the rate of proteins discarded via the ER-associated degradation. In addition, ER stress can block the mTORC1 pathway which is essential in the response to the anabolic stimulus of nutrients and contractile activity thereby participating to the well-known anabolic resistance state in skeletal muscle during ageing. As exercise increases the expression of several chaperones, it could anticipate or restore the loss of unfolded protein response components with age and thereby reduce the level of ER stress. This hypothesis has not been tested yet but it could be a new mechanism behind the beneficial effects of exercise in the elderly not only for the preservation of muscle mass but also for the regulation of whole body metabolism.

## Introduction

Skeletal muscle is vital to life as it provides the mechanical power for locomotion, posture and breathing. Beyond these vital functions, skeletal muscle also plays an essential role in the regulation of whole body metabolism, e.g., glucose homeostasis. Although progressive loss of muscle mass with age seems unavoidable, it is critical for older people to keep the highest mass as possible. This loss of muscle mass, also called sarcopenia, becomes increasingly important at a socio-economically point of view due to the augmenting proportion of elderly in the contemporary society (Janssen and Ross, 2005). A reduction of skeletal muscle tissue reduces the ability of the organism to cope with changing metabolic demands and leads to and/or reinforces pathologies such as diabetes or obesity. Prevalence figures vary from roughly 7% to 70% of elderly suffering from sarcopenia, depending on age, sex, health status, comorbid conditions and others (Malafarina et al., 2012). The rate of skeletal muscle loss is estimated at 8% per decade from the 4th until 7th decade, and after 70 years, muscle loss would occur at a speed of 15% per decade. Loss of strength is estimated to be even larger (Malafarina et al., 2012). Counteracting sarcopenia and consequently increasing the independence of our elderly population is one of the major challenges at the moment.

## Sarcopenia and anabolic resistance

The term sarcopenia was first proposed in 1989 by Irwin Rosenberg and is derived from the Greek “sarx,” meaning flesh and “penia,” meaning loss (Rosenberg, 1989). Originally, sarcopenia referred thus to the loss of muscle mass associated with ageing. Since then, the significance of this term has been extended to the age-related loss of muscle strength as well. The skeletal muscle mass index (SMI) allows the quantification of the level of sarcopenia (Baumgartner et al., 1998). It is obtained by dividing appendicular skeletal muscle mass (ASM), evaluated by DEXA, by body height squared (ASM/height^2^). According to this definition, individuals presenting an ASM/height^2^ ratio between −1 and −2 standard deviations of the gender-specific mean value of young adults are categorized as having class I sarcopenia. Individuals with an ASM/height^2^ ratio below −2 standard deviations are categorized as having class II sarcopenia. Another definition of sarcopenia uses a percentage of the skeletal muscle index (SMI% = total muscle mass/body mass × 100) (Janssen et al., 2002). Other strategies have also been used to develop cut-points to distinguish between sarcopenic older adults and older adults with a relatively healthy muscle mass (Janssen and Ross, 2005).

The etiology of sarcopenia is rather complex since it involves non exhaustively: (1) alterations of the central and peripheral nervous system: α-motoneuron degeneration and muscle fiber denervation; (2) changes in hormones levels: decrease in testosterone, androgens, estrogens, growth hormone, insulin-like growth factor I and increase in myostatin; (3) nutritional factors: anorexia of ageing, vitamin D deficiency; (4) the immunological system: decrease in interleukin-1β, interleukin-6 and tumor necrosis factor levels; (5) skeletal muscle redox status: increased reactive oxygen species production, altered mitochondrial function and increased oxidative stress and; (6) a decrease in physical activity (Meng and Yu, 2010; Narici and Maffulli, 2010). Although the individual contribution of each factor is very difficult to establish, it may be generally stated that sarcopenia results from a mismatch between protein synthesis and protein degradation. In humans, basal protein synthesis and breakdown have been found to be unaffected or only slightly modified with ageing (Welle et al., 1995; Balagopal et al., 1997; Volpi and Rasmussen, 2000; Volpi et al., 2001; Cuthbertson et al., 2005). However, differences in protein synthesis between young and older individuals do exist in response to feeding and exercise, with older people showing a blunted response to anabolic stimuli. This phenomenon has been called anabolic resistance and seems to hold a major role in the development of sarcopenia (Rennie, 2009; Rennie et al., 2010; Breen and Phillips, 2013). Compared to young controls, older people show a lower increase in muscle protein synthesis in response to amino acid feeding under insulin clamping and to an acute bout of exercise (Cuthbertson et al., 2006). Likewise, despite the absence of differences in breakdown in the basal state, important differences have been recently discovered in response to feeding. The inhibition of proteolysis by insulin in response to feeding and activation of anabolic signaling pathway are blunted in older individuals compared with young adults (Wilkes et al., 2009). At a molecular point of view, the protein kinase B (PKB)/mammalian target of rapamycin complex 1 (mTORC1) pathway shows a blunted increase in phosphorylation after a meal in old compared with young people.

**Figure 1:**
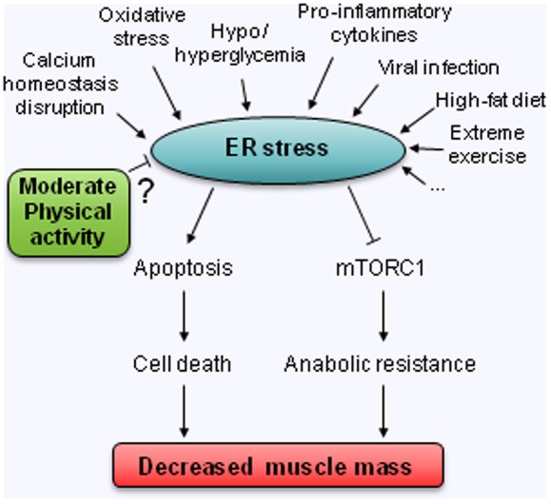
**Hypothetical model for the involvement of ER stress in the decreased muscle mass observed with age. ? means untested relation**.

Whereas the origin of sarcopenia is obviously multifactorial, in the present review, it was deliberately chosen to evaluate the likely contribution of one specific cellular stress, namely the endoplasmic reticulum (ER) stress (Figure 1). It is proposed that ER stress can: (1) directly impact muscle mass as one fate of prolonged and unresolved ER stress is cell death and; (2) indirectly create a state of anabolic resistance by inhibiting the mTORC1 pathway.

## Endoplasmic reticulum stress

The ER is a membrane-bound cell organelle responsible for the folding, processing and trafficking to the cell surface of all secretory and integral membrane proteins. It is also a critical site for the quality control of proteins, calcium homeostasis and cholesterol and lipid biosynthesis. It contains a netlike membranous network that extends throughout the cytoplasm and can be connected with the nuclear membrane. It can therefore sense and transmit signals that originate in any cellular subcompartment (Kaufman et al., 2002). Protein maturation, folding and trafficking is of a huge strategical importance to cellular functioning and disturbances in ER homeostasis can impair its functioning. As a non exhaustive list, viral infection (Isler et al., 2005), abnormal calcium regulation (Pyrko et al., 2007), various mutations (Chen et al., 2013) as well as high-fat feeding (Deldicque et al., 2010) have all been found to disrupt ER homeostasis, thereby creating ER stress. These perturbations can lead to the accumulation of unfolded proteins and protein aggregates in the lumen of the ER which can be injurious to the cell. In a non-pathological situation cells ensure correct protein folding using a combination of molecular chaperones, foldases, and lectins. When these fail to restore the protein to its biological active structure the incorrectly processed proteins are targeted to the ER associated degradation (ERAD) or to degradation by autophagy (Kaufman et al., 2002; Ishida and Nagata, 2009; Verfaillie et al., 2010). Continued accumulation of incorrectly folded proteins can also trigger the unfolded protein response.

The unfolded protein response is a signaling pathway primarily aiming to protect the cellular integrity by restoring ER folding capacity by chaperone induction, attenuating protein translation, and degrading misfolded proteins (Wu and Kaufman, 2006; Chakrabarti et al., 2011). The unfolded protein response is composed of three main branches, each of them being activated by a specific stress transducer: protein kinase R-like ER protein kinase (PERK), activating transcription factor 6 (ATF6) and inositol-requiring enzyme 1 alpha (IRE1α). These stress transducers have a ER-luminal part that sense the protein-folding environment, and a cytoplasmic part that interact with the transcriptional and/or translational apparatus (Ron and Walter, 2007). In the basal state, they are all associated with the chaperone BiP also called glucose-regulated protein 78 (GRP78). Upon accumulation of unfolded/misfolded proteins each transducer can disassociate from BiP, which results in their activation (Gething, 1999). ATF6 and PERK are thought to be activated before IRE1α, consistent with the signals these effectors are transducing. The former two mainly promote ER adaptational responses to folding errors and the latter has a more dual role consisting of transmitting both survival and pro-apoptotic signals (Chakrabarti et al., 2011). For detailed description of the unfolded protein response, the reader is referred to the following reviews: Schroder and Kaufman (2005); Wu and Kaufman (2006); Ron and Walter (2007); Chakrabarti et al. (2011); Walter and Ron (2011); Maurel and Chevet (2013).

When ER stress becomes chronic and the capacity of the unfolded protein response to face this stress is exceeded, inflammatory processes will be activated (Zhang and Kaufman, 2008a). ER stress-induced inflammation is mainly mediated by Jun NH2-terminal kinase (JNK) and nuclear factor-kappa B (NF-κB). JNK activation, which occurs through IRE1α-dependent signaling, induces the expression of inflammatory genes by phosphorylating AP1 (transcription activator protein1) (Zhang and Kaufman, 2008a). NF-κB-dependent transcription is increased by two ways during ER stress. First, the level of the inhibitor of NF-κB (IκB), which has a shorter half-life than NF-κB, is reduced when protein translation is attenuated thereby changing the stoichiometric ratio of NF-κB:IκB, freeing NF-κB from its inhibitor and allowing NF-κB to translocate to to the nucleus. Secondly, the IRE1α-tumor necrosis factor receptor associated factor 2 (TRAF2) complex recruits IκB kinase (IKK) that phosphorylates IκB and leads to its degradation (Hu et al., 2006; Zhang and Kaufman, 2008a), thereby activating the inflammatory response.

## Endoplasmic reticulum stress, apoptosis, and cell death

**Figure 2:**
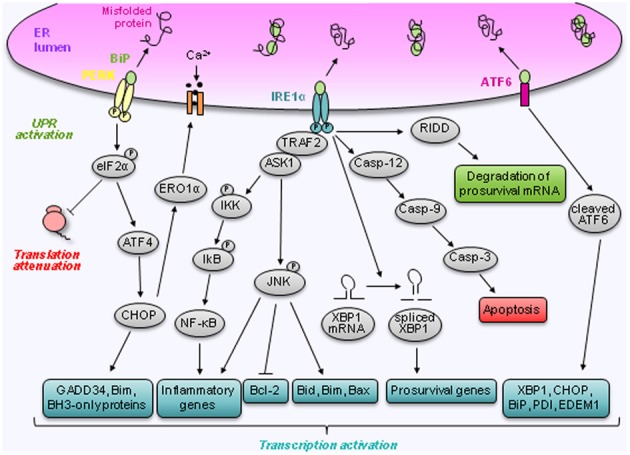
**Regulation of prosurvival and apoptotic pathways by ER stress.** Cells cope with ER stress by activating the unfolded protein response. This response is mediated via the dissociation of BiP from three ER transmembrane proteins IRE1α, PERK, and ATF6. Following dissociation of BiP, IRE1α becomes activated and induces splicing of XBP1 mRNA to XBP1s. IRE1α also activates JNK via TRAF2 and ASK1. Furthermore, activation of IRE1α has been linked to downstream NF-κB activation and RIDD, which can lead to the degradation of prosurvival mRNA. Finally, IRE1α controls the activation of the caspases signaling pathway. Like IRE1α, PERK becomes activated following BiP dissociation. Active PERK mediates its response via phosphorylation of eIF2α leading to a translational block and cap independent translation of ATF4. ATF4 induces CHOP which has multiple downstream targets that stimulate apoptosis and cell death. Following BiP dissociation, ATF6 is transported to the Golgi where it is cleaved into an active transcription factor. ATF6 regulates the expression of several genes involved in the unfolded protein response such as XBP1, CHOP, BiP, PDI, and EDEM1.

Ultimately, uncontrolled and excessive ER stress will lead to apoptosis and cell death (Ron and Walter, 2007; Zhang and Kaufman, 2008b). Initially, Schroder and Kaufman (2005) distinguished two different mechanisms by which ER stress induces apoptosis: the intrinsic and the extrinsic pathways. According to these authors, the intrinsic pathway responds to intracellular insults, e.g., DNA damage, whereas the extrinsic pathway responds to extracellular stimuli and is triggered by self-association of cell surface receptors, recruitment of caspases, mainly caspase-8, and initiation of a caspase cascade. Since then, additional mechanisms have been discovered and involve, amongst others, IRE1α, regulated IRE1-dependent decay of mRNAs (RIDD), C/EBP homologous protein (CHOP) also known as growth arrest and DNA damage 153 (GADD 153), the Bcl-2 family members (Bak/Bax), caspase-12 and JNK (Logue et al., 2013) (Figure 2).

### IRE1α

Thanks to its RNase activity, IRE1α can splice a 26 nucleotide intron from X-binding protein 1 (XBP1) mRNA generating a transcription factor called spliced XBP1 (XBP1s) (Yoshida et al., 2001). XBP1s has a diverse range of target genes which share the common aim of short term adaptation and ultimately restoration of ER function. Recent reports also suggest XBP1s signaling may be able to modulate apoptotic signaling by regulating Bcl-2 levels (Gomez et al., 2007; Kurata et al., 2011) but it remains currently unknown whether XBP1s can modulate Bcl-2 family member expression in response to ER stress. Overexpression of IRE1α in human embryonic kidney cells has been reported to induce death indicating that IRE1α could activate pro-apoptotic signaling components (Wang et al., 1998). Indeed the recruitment of TRAF2 to IRE1α has been linked to several pro-apoptotic pathways the most well defined being the IRE1α-TRAF2-JNK axis (Urano et al., 2000). This axis can thus either trigger inflammation by activating AP-1 (Zhang and Kaufman, 2008a) or cell death by modulating Bcl-2 family members function (Logue et al., 2013).

### Ridd

The RNase activity of IRE1α has recently been linked to a process referred to as regulated IRE1α-dependent decay of mRNAs (RIDD) (Hollien and Weissman, 2006; Han et al., 2009). While this process is reliant upon IRE1α RNase activity it is distinct from XBP1 splicing and is reported to selectively target and degrade mRNAs encoding secretory proteins involved in protein folding within the ER (Logue et al., 2013). Initial activation of RIDD would be expected to aid cell survival by reducing the protein load on the ER. However, prolonged RIDD signaling has been reported to correlate with increased apoptosis (Han et al., 2009). The switch between anti-apoptotic XBP1s signaling and pro-apoptotic RIDD may be dependent upon the conformational state of IRE1α (Han et al., 2009). IRE1α-mediated RIDD activation has only been recently discovered and further studies are required to identify RIDD targets and the mechanisms controlling its activation.

### Chop

CHOP upregulation is a common point of convergence for all 3 arms of the unfolded protein response with binding sites for ATF6, ATF4, and XBP1s present within its promoter (Logue et al., 2013). CHOP signaling is thought to mediate cell death signaling by firstly altering the transcription of genes involved in apoptosis and oxidative stress and secondly by relieving PERK-mediated translational inhibition, thereby enhancing the translation of pro-apoptotic proteins (Oyadomari and Mori, 2004). Transcriptional targets of CHOP include BH3-only members of the Bcl-2 family (and more particularly Bim, Puma, and Noxa), ER oxidoreductin 1 alpha (ERO1α) and tibbles-related protein 3 (TRB3), all being able to regulate cell death (Logue et al., 2013). The combination of increased BH3-only protein expression and repression of anti-apoptotic proteins such as Bcl-2 shifts the balance in favor of apoptosis permitting Bax-Bak homo-oligomerization and mitochondrial outer membrane permeabilization causing cytochrome c release and subsequent apoptosome formation. Overexpression of Bcl-2 reduces the loss of mitochondrial membrane potential and protects cells against ER stress underscoring the importance of mitochondrial mediated signals in the propagation of ER stress-induced apoptosis (Heath-Engel et al., 2008).

### Caspases

The caspase family of cysteine proteases is a key mediator of programmed cell death (Thornberry, 1998). Murine caspase-12 (caspase-4 in human) is an initiator caspase and a central player in ER-induced apoptosis (Szegezdi et al., 2006). Once activated, caspase-12 translocates from the ER to the cytosol where it cleaves caspase-9, which in turn induces the cleavage of the executioner caspase, caspase-3, in a cytochrome c-independent manner, and activation of the rest of the apoptotic pathway.

### microRNAs

The regulation of ER stress-induced death pathways by microRNAs is a recent area of research with studies indicating miRNAs can either directly modulate the ER stress response or themselves be regulated by ER stress (Logue et al., 2013). For example, in hepatocellular carcinoma cells, miR-122 overexpression downregulated ER stress responses (Yang et al., 2011) whereas ER stress-mediated downregulation of miR-221/222 was associated with resistance to cell death (Dai et al., 2010). Direct regulation of miRNA expression by ER stress sensors, particularly PERK, has been reported and may regulate the subtle balance that exists between pro-and anti-apoptotic signaling during ER stress. PERK-mediated induction of miR-30c-2^*^ has been linked to a decrease in XBP1 mRNA reducing pro-survival signaling and favoring cell death (Byrd et al., 2012). In the same way, PERK-mediated repression of miR-106b-25 has been reported to result in increased Bim expression, which is essential for ER stress-induced apoptosis, and apoptosis itself (Gupta et al., 2012). Conversely, miR-211 was identified to be a PERK target and to repress CHOP expression on a short-term, thereby supporting a pro-survival response. Upon sustained ER stress, miR-211 expression was silenced, permitting CHOP accumulation and induction of the pro-apoptotic response (Chitnis et al., 2012). All together, these results suggest that miRNA regulation help shift the balance between survival and cell death during ER stress (Logue et al., 2013).

## Endoplasmic reticulum stress and ageing

The ageing process contains lots of characteristics indicating that ER stress could be activated, e.g., increased oxidative stress and accumulation of harmful protein modifications, misfolding and aggregation of proteins, disturbances in calcium homeostasis and impairment in global protein synthesis (Finkel and Holbrook, 2000; Tavernarakis, 2008; Puzianowska-Kuznicka and Kuznicki, 2009). In addition, the protein cleansing system becomes impaired during ageing due to the decline in autophagic and proteasomal degradation (Vernace et al., 2007; Salminen et al., 2011). All these age-related changes imply that the efficient function of protein quality systems is compromised during ageing. The capital role of ER stress in many ageing-related neurodegenerative diseases such as Parkinson's disease, amyothrophic lateral sclerosis and Alzheimer disease, witnesses the importance of the ER during ageing. But not only is the brain affected by ER stress during ageing, the efficiency of the unfolded protein response has also been found to be reduced in the liver, the lung, and the heart. Those changes in the unfolded protein response with age are presented and discussed in details in (Naidoo, 2009a,b; Salminen and Kaarniranta, 2010; Brown and Naidoo, 2012). In the next paragraphs the most important alterations in the unfolded protein response with age are summarized.

### Decrease in chaperones content

Recent reports have indicated that ER stress increases during the lifespan (Naidoo, 2009b). Key components of the unfolded protein response display reduced expression and activity with age resulting in a decreased ability to cope with ER stress (Ogata et al., 2009). ER chaperones and folding enzymes are crucial to correctly fold proteins. This is of vital importance for the biological function and cellular survival of the proteins. Key chaperones and folding enzymes include BiP, 94 kDa glucose-regulated protein (GRP94), lectins such as calnexin and calreticulin, thiol-disulfide oxidoreductases such as protein disulfide isomerase (PDI), also known as ER resident protein 58 (ERp58), and ERp57.

#### BiP/GRP78

As described previously, BiP concentration in the lumen of the ER is crucial for correct protein folding (Gething, 1999). However BiP expression decreases during the lifespan in murine brain (Paz Gavilan et al., 2006; Hussain and Ramaiah, 2007) and liver (Erickson et al., 2006; Hussain and Ramaiah, 2007; Nuss et al., 2008).

#### Lectins

Calnexin and calreticulin are proteins responsible for glycoprotein quality control in the ER. Calnexin is a transmembrane protein and calreticulin is the soluble luminal homolog. Both have been found to decline with age (Naidoo, 2009b; Ogata et al., 2009). In aged rat liver (Erickson et al., 2006) and hippocampus (Paz Gavilan et al., 2006), calnexin expression is downregulated by about one third. A decrease in calnexin expression has been suggested to sensitize cells to apoptosis through accumulation of GD3, a ganglioside acting like an apoptotic mediator (Tomassini et al., 2004). Accumulation and translocation of GD3 to the mitochondria induces caspases activation, release of apoptotic factors, and disturbs its membrane potential (Garcia-Ruiz et al., 2000).

#### Protein disulfide isomerase

PDI catalyzes native disulfide bond formation and its expression has been found to be reduced by about 50% in the hippocampus of old compared to young rats (Paz Gavilan et al., 2006). Another study in rat liver reported a similar decrease of ERp55 and ERp57 protein expression, which are PDI-related proteins that protect the cell from oxidative injury (Erickson et al., 2006). In addition, BiP, PDI, and calreticulin are affected by reactive oxygen species and this damage is associated with a reduced enzyme activity (Nuss et al., 2008). The progressive oxidation of ER chaperones forms probably an important change affecting the unfolded protein response with age (Naidoo, 2009a).

### Increase in pro-apoptotic markers

Another important change with age is an increase in pro-apoptotic markers in case of ER stress (Naidoo, 2009a; Torres-Gonzalez et al., 2012). An increased level of CHOP has been found in aged mouse cortex (Naidoo et al., 2008) as well as in aged rat hippocampus, cortex, cerebellum, lung, liver, kidney, heart, and spleen (Paz Gavilan et al., 2006; Hussain and Ramaiah, 2007). A decrease in CHOP has important consequences since it mediates apoptosis in response to ER stress and elevated CHOP sensitizes cells to oxidative insults (Ikeyama et al., 2003). Elevated CHOP levels could form an explanation for the increased sensitivity to oxidative damage with age described earlier. Next to increased expression of CHOP, ageing is accompanied with an increased activity of caspases (Naidoo, 2009b). The latter's play a central role in programmed cell death by participating in a cascade triggered by pro-apoptotic signals that culminates in the cleavage of targeted proteins. This cascade ultimately leads to disassembly of the cell (Thornberry, 1998). In aged rat hippocampus, caspase-12 is activated in response to ER stress but not in young animals (Paz Gavilan et al., 2006). The same results were obtained in mice cerebral cortex while sleep deprivation-induced ER stress was investigated (Naidoo et al., 2008). Finally phosphorylation of JNK is increased with age (Hussain and Ramaiah, 2007), which results in a higher apoptotic rate due to, amongst others, inhibition of the anti-apoptotic Bcl-2 and activation of the translocation of Bax to the mitochondrial membrane (Gao et al., 2005).

## Endoplasmic reticulum stress in skeletal muscle

ER stress has been widely studied in pancreatic islets, liver, and adipose tissue. Despite the fact that skeletal muscle is primarily responsible for glucose disposal and therefore intimately related to disease states like diabetes and obesity, this tissue has been neglected and much less information exists about ER stress in skeletal muscle in comparison with the other metabolic organs (see Deldicque et al., 2012 for a review on the topic). Even though it has a restricted secretory function, skeletal muscle is interesting with respect to the unfolded protein response because it contains an extremely extensive network of specialized ER called the sarcoplasmic reticulum. Since it is essential to maintain the optimal calcium concentration in the lumen of the sarcoplasmic reticulum for the regulated release of calcium from sarcoplasmic reticulum during contraction in skeletal muscle, any disturbance in the ER could impair muscle contraction (Deldicque et al., 2012).

In skeletal muscle, ER stress was initially observed in myopathies, such as myotonic dystrophy Type 1 (Ikezoe et al., 2007) and inclusion body myositis (Nogalska et al., 2006). Nowadays evidence supporting the existence of ER stress in non-pathological skeletal muscle is accumulating (Deldicque et al., 2012). As in other organs, ER stress in skeletal muscle can be caused by several nutritional insults, such as imbalance in glucose concentrations or high-fat feeding. High glucose incubation *in vitro* (Srinivasan et al., 2009) as well as high fat intake *in vivo* (Deldicque et al., 2010, 2013) activated the unfolded protein response in skeletal muscle cells. However, increasing energy intake by increased fat ingestion did not result in an activation of the unfolded protein response in human skeletal muscle (Deldicque et al., 2011). Neither was the case in skeletal muscle of fasted rats for up to 3 days (Ogata et al., 2010). More and more evidence accumulate showing that the unfolded protein response is activated by contractile activity and inactivity in skeletal muscle. Exhaustive endurance exercise activates the unfolded protein response in mice (Wu et al., 2011) and human skeletal muscle (Kim et al., 2011). Immobility triggers the unfolded protein response in humans as well (Alibegovic et al., 2010), indicating that extremely low or high level of contractile activity activates ER stress and the unfolded protein response. Not surprisingly, repeated bouts of moderate endurance exercise seem rather protective against subsequent ER stress as this kind of exercise training increases the expression of several chaperones in high-fat fed mice (Deldicque et al., 2013).

## Ageing alters the unfolded protein response in skeletal muscle

With age, many of the key components of the unfolded protein response such as the chaperones and enzymes display reduced expression and activity resulting in a dysfunctional ER and the development of cellular stress (Naidoo, 2009a). As mentioned above, ER stress has been implicated in many ageing related neurodegenerative diseases but does it specifically impact skeletal muscle during ageing? The number of studies dealing with ER stress and/or the unfolded protein response in skeletal muscle during ageing is very scarce. In skeletal muscle of 32-month-old rats, the expression of specific chaperones such as ERp29, HSP70, and calreticulin was decreased compared to 6-month-old rats while at the same time ER stress and apoptosis markers were increased (Ogata et al., 2009). In another study looking at the effect of denervation and ageing, CHOP protein expression and XBP1s mRNA level were much higher whereas BiP protein expression was halved in aged rats compared to young controls (O'Leary et al., 2013). In response to denervation, CHOP and XBP1s expressions increased in both groups but the level reached in young animals was still below that of old animals. Although data in old skeletal muscle are limited, those first observations confirm the results obtained in other tissues, e.g., a decrease in chaperones content and an increase in apoptosis markers with age.

To the best of my knowledge, there is no report dealing with ER stress and/or the unfolded protein response during ageing in human skeletal muscle. However it would not be surprising to find a decreased capacity to face ER stress. Ageing is often accompanied with a high caloric nutrients intake coupled to a reduced physical activity, a reduced insulin sensitivity and a decreased capacity of the oxidative metabolism, all known to be exacerbated by ER stress and to affect skeletal muscle to a large extend.

## Does endoplasmic reticulum stress induce anabolic resistance thereby exacerbating sarcopenia?

As described above, a major cause of the reduction in muscle mass with ageing is anabolic resistance, which is defined as a blunted response to hypertrophic stimuli such as exercise and nutrition. At a molecular level, the activation of the mTORC1 pathway seems to be impaired with age (Rennie, 2009). As the ER acts as a nutrient sensor and links nutrient sensing to cellular signaling through the unfolded protein response (Mandl et al., 2009), we recently tested the hypothesis that anabolic resistance can be partially due to disturbance in the ER homeostasis (Deldicque et al., 2011). In that study, we sought to determine whether ER stress could induce anabolic resistance in C2C12 muscle cells (Deldicque et al., 2011). Consistent with this hypothesis, low levels of ER stress were sufficient to prevent the activation of mTORC1 by leucine. The inability to activate mTORC1 was not due to a lack of leucine transport, but rather to the ER stress-induced decrease in basal PKB phosphorylation resulting in PRAS40 hypophosphorylation and inhibition of mTORC1. In C2C12 muscle cells, ER stress seems to impair mTORC1 rather than vice versa (Ozcan et al., 2008; Kang et al., 2011). Hyperactivation of mTORC1 by insulin for 6 h or 24 h did not trigger the unfolded protein response whereas tunicamycin activated the unfolded protein response before S6K1 phosphorylation decreased, suggesting that in C2C12 muscle cells the induction of ER stress precedes the impairment in mTORC1 activity (Deldicque et al., 2010). Therefore, in the present case, ER stress is a contributor to the impairment on the mTOR pathway rather than the consequence. Whether the blunting response of leucine on the mTORC1 pathway results in a decreased protein synthesis and finally to a loss of muscle mass *in vivo* has not been tested yet but this study in cell cultures has the merit to highlight ER stress as a potential candidate to the decrease in muscle mass observed in several conditions such as in ageing (Cuthbertson et al., 2005), immobilization (Glover et al., 2008); and high-fat feeding/obesity (Sitnick et al., 2009). Knowing that: (1) ageing is characterized by a decreased capacity to face ER stress as well as by a loss of muscle mass and, (2) ER stress impairs the mTORC1 pathway, thereby favoring anabolic resistance, it is tempting to bring those 2 observations into the assumption that ER stress contributes to anabolic resistance leading to sarcopenia. Although each separate assertion has been documented in the literature, the contribution of ER stress to anabolic resistance induced-sarcopenia remains purely hypothetical.

## Perspectives

Further research will be required to confirm the effective involvement of ER stress in anabolic resistance induced-sarcopenia and to determine the molecular mechanisms linking both events. Also, the difference between eugeric and pathogeric ageing (Finch, 1972) should be taken into consideration in future studies. Pathogeric ageing is probably characterized by a certain level of ER stress in skeletal muscle due to the increase in factors known to trigger ER stress such as high lipids concentrations or increased oxidative stress. Eugeric ageing is not accompanied by those changes and the probability to induce ER stress in skeletal muscle is rather small. However, a decrease in chaperones seems inevitable with ageing, even during eugeric ageing, which reduces the capacity of the cell to face stressful situations. One could postulate that pathogeric ageing is characterized by an increased activation of ER stress and a decreased unfolded protein response whereas eugeric ageing by a decreased response only. As a result ER stress in skeletal muscle would be less important in eugeric ageing than in pathogeric ageing but would still be higher than at middle age.

In a preventive and/or therapeutic perspective, it would be useful to find out which factors trigger ER stress in skeletal muscle during ageing to counteract this stress optimally. Another strategy would be to anticipate or to restore the loss of unfolded protein response components with age. In this perspective, exercise could be a useful tool as it increases the expression of several chaperones and thereby could reduce the level of ER stress. This hypothesis has not been tested yet but it could be a new mechanism behind the beneficial effects of exercise in the elderly not only for the preservation of muscle mass but also for the regulation of whole body metabolism.

## Conclusions

The number of publications dealing with ER stress and its downstream signaling, the unfolded protein response, has increased exponentially these last years, underlying the importance of this cellular stress in many different tissues and several pathologies. In the present report, a novel role for ER stress is proposed, namely a contribution to the well-known anabolic resistance-induced sarcopenia. In addition to stimulating apoptosis, it is suggested that ER stress negatively regulates protein balance by inhibiting the mTORC1 pathway, and thereby contribute to the loss of muscle mass with age.

### Conflict of interest statement

The author declares that the research was conducted in the absence of any commercial or financial relationships that could be construed as a potential conflict of interest.
